# Sugar-fermenting yeast as an organic source of carbon dioxide to attract the malaria mosquito *Anopheles gambiae*

**DOI:** 10.1186/1475-2875-9-292

**Published:** 2010-10-25

**Authors:** Renate C Smallegange, Wolfgang H Schmied, Karel J van Roey, Niels O Verhulst, Jeroen Spitzen, Wolfgang R Mukabana, Willem Takken

**Affiliations:** 1 Laboratory of Entomology, Wageningen University, P.O. Box 8031, 6700 EH, Wageningen, The Netherlands; 2 International Centre of Insect Physiology and Ecology, P.O. Box 30772 - 00100, GPO, Nairobi, Kenya; 3 School of Biological Sciences, University of Nairobi, P.O. Box 30197 - 00100 GPO, Nairobi, Kenya

## Abstract

**Background:**

Carbon dioxide (CO_2_) plays an important role in the host-seeking process of opportunistic, zoophilic and anthropophilic mosquito species and is, therefore, commonly added to mosquito sampling tools. The African malaria vector *Anopheles gambiae sensu stricto *is attracted to human volatiles augmented by CO_2_. This study investigated whether CO_2_, usually supplied from gas cylinders acquired from commercial industry, could be replaced by CO_2 _derived from fermenting yeast (yeast-produced CO_2_).

**Methods:**

Trapping experiments were conducted in the laboratory, semi-field and field, with *An. gambiae s.s*. as the target species. MM-X traps were baited with volatiles produced by mixtures of yeast, sugar and water, prepared in 1.5, 5 or 25 L bottles. Catches were compared with traps baited with industrial CO_2_. The additional effect of human odours was also examined. In the laboratory and semi-field facility dual-choice experiments were conducted. The effect of traps baited with yeast-produced CO_2 _on the number of mosquitoes entering an African house was studied in the MalariaSphere. Carbon dioxide baited traps, placed outside human dwellings, were also tested in an African village setting. The laboratory and semi-field data were analysed by a χ^2^-test, the field data by GLM. In addition, CO_2 _concentrations produced by yeast-sugar solutions were measured over time.

**Results:**

Traps baited with yeast-produced CO_2 _caught significantly more mosquitoes than unbaited traps (up to 34 h post mixing the ingredients) and also significantly more than traps baited with industrial CO_2_, both in the laboratory and semi-field. Adding yeast-produced CO_2 _to traps baited with human odour significantly increased trap catches. In the MalariaSphere, outdoor traps baited with yeast-produced or industrial CO_2 _+ human odour reduced house entry of mosquitoes with a human host sleeping under a bed net indoors. *Anopheles gambiae s.s*. was not caught during the field trials. However, traps baited with yeast-produced CO_2 _caught similar numbers of *Anopheles arabiensis *as traps baited with industrial CO_2_. Addition of human odour increased trap catches.

**Conclusions:**

Yeast-produced CO_2 _can effectively replace industrial CO_2 _for sampling of *An. gambiae s.s*.. This will significantly reduce costs and allow sustainable mass-application of odour-baited devices for mosquito sampling in remote areas.

## Background

Carbon dioxide (CO_2_), a major constituent of vertebrate breath, plays an important role in the host-seeking process of mosquitoes [1-6]. Therefore, the compound is commonly added to traps used for mosquito surveillance [7-9]. Among malaria vectors, opportunistic, zoophilic as well as anthropophilic mosquito species are affected by CO_2 _[2,4,6,10-13]. In *Anopheles gambiae sensu stricto*, an important vector of human malaria in sub-Saharan Africa and considered to be highly anthropophilic [14], CO_2 _augments the attractiveness of human odour [6,12] and it is an essential cue to lure the female mosquitoes into the vicinity of mosquito traps [5,13].

Even though CO_2 _has a positive effect on the number of mosquitoes that are caught by suction traps, in resource-poor areas, like sub-Saharan Africa, it is hard to obtain CO_2 _sources that are reliable, cheap, easy to manage and durable. Propane-powered traps that produce CO_2 _[15] are difficult to obtain, heavy and expensive. The same is true for industrially-acquired CO_2_, which, packaged in steel cylinders, has the advantage that the release rate of CO_2 _can be regulated, but leakage at the connections may occur. In addition, flow meters may be costly and sensitive to dust and high humidity. Dry ice, an alternative source of CO_2_, is cheap and easier to handle than pressurized CO_2 _cylinders, but is difficult to obtain and transport in the tropics, besides the need for replenishment on a regular basis. Moreover, dry ice has the disadvantage that the release rate of CO_2 _is highly variable and diminishes over time [2,16].

Saitoh *et al *[16] developed an easy and cheap method to produce CO_2 _by using a yeast-sugar solution in plastic bottles. Under anaerobic conditions, yeast (synonym for strains of *Saccharomyces cerevisiae *or baker's yeast) converts sugar into CO_2 _and ethanol [17-20]. In Japan, traps baited with yeast-generated CO_2 _caught higher numbers of *Aedes *and *Culex *spp. than unbaited traps. The objective of the present study was to investigate, under laboratory, semi-field and African field conditions, whether this method is valuable to lure *An. gambiae s.s*. females towards suction traps, as an alternative for industrial-acquired CO_2_.

## Methods

### Mosquitoes

Female mosquitoes used for the laboratory experiments were collected from a culture of *Anopheles gambiae s.s*. (hereafter referred to as *An. gambiae*) (Suakoko strain) kept at Wageningen University, The Netherlands. The culture has been reared by blood-feeding on human arms since 1988. Larvae were kept in tap water and fed on Tetramin^®^ baby fish food. Pupae were collected daily and transferred to 30 cm cubic gauze cages for emergence. Adult mosquitoes were kept at 27°C, 80% RH and a photo:scotophase of 12:12 h, respectively. A 6% glucose solution was provided *ad libitum *on filter paper.

The semi-field experiments were conducted using the Mbita strain of *An. gambiae*. The mosquitoes have been reared under ambient climatic conditions at insectaries belonging to the Thomas Odhiambo campus of the International Centre of Insect Physiology and Ecology (ICIPE) located at Mbita Point, western Kenya, since 2001. Adult insects were kept in 30 cm cubic gauze cages and provided with a 6% glucose solution *ad libitum*. Blood feeding took place on human arms. Larvae were kept in filtered water from Lake Victoria and fed on Tetramin^®^ baby fish food. Upon pupation, insects were transferred to adult cages for emergence.

The age of the female mosquitoes used for the laboratory experiments was 5-8 days; the *An. gambiae *females used for the semi-field experiments were 3-7 days old. The females, previously not blood-fed, were randomly collected from their cage and placed in a release cage (d = 8 cm, h = 20 cm in the laboratory experiments or d = 11-13 cm, h = 15 cm in the semi-field experiments) 16 (laboratory) respectively 8 (semi-field) h before the experiments were started. To prevent dehydration the mosquitoes were offered water-moistened cotton wool on top of the release cage.

### Traps

Mosquito Magnet-X counter flow geometry traps (MM-X; American Biophysics Corp., USA, [21], see also [22,23]), were suspended from metal or wooden stands, with the odour outlet 15 cm above ground level [12,13]. The bullet-shaped cartridges within the lower end of the odour outlet tube of the traps were removed. The electric ventilators in the MM-X traps operated on 12 V batteries. During the experiments performed in the MalariaSphere [24] also CDC miniature light traps (Model 512; John W. Hock Company, USA, [25]) were used. These traps were run on 6 V batteries (Gaston Battery Industrial Ltd, China). After removing the caught mosquitoes, each trap was cleaned with 10% ethanol.

### Odour stimuli

**Figure 1:**
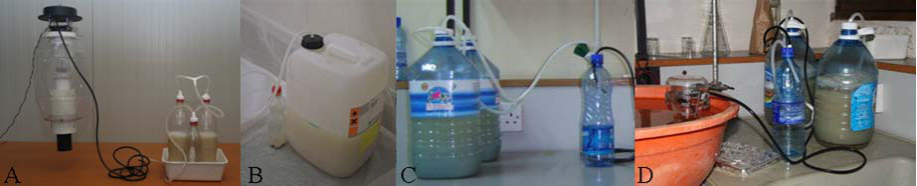
**Pictures showing the different setups used to apply the yeast-sugar solutions and to measure the CO_2 _production**. A. Two 1.5 L bottles; B. One 25 L container; C. Two 5 L bottles; D. CO_2 _production measurement.

Yeast-produced carbon dioxide was produced by mixing dry yeast (Dr. Oetker, The Netherlands, used in the laboratory experiments carried out in Wageningen or Angel Yeast Co. Ltd., China, used in the semi-field and field experiments in Kenya), sugar (Van Gilse Kristalsuiker, Suiker Unie, The Netherlands, in the laboratory experiments or Sony Sugar, South Nyanza sugar Co. Ltd., Kenya, in the (semi-)field experiments) and tap water [16] in two plastic bottles of 1.5 L or 5 L, connected with each other by silicon tubing, or one plastic container of 25 L. Mixing took place 1-1½ h before mosquitoes were released, at ambient temperature, until the dry yeast was dissolved. No additional stirring or mixing took place during the experiments. A 0.5 L respectively 1 L bottle was put in between the 1.5 L respectively 5 L bottles with the mixtures and the MM-X trap to prevent foam produced by the mixtures entering the trap (Figure 1A-C). Holes were drilled into the original screw caps of the bottles and into the side of the small bottles; silicon tubing (Ø 7 mm; Rubber B.V., The Netherlands) fitted through these holes to connect the bottles. The smaller bottle was connected to the MM-X trap using the original MM-X tubing (micron filter and orifice removed) and the Luer connection at the underside of the trap's top lid. The connections were sealed by Teflon tape and held under water to check for leakage. Several combinations of bottle size and amount of yeast, sugar and water were used. The carbon dioxide output was estimated by measuring the volume of water displaced from a submerged measuring cylinder (Table 1). For this purpose, the tubing that was attached to the MM-X traps during the mosquito trapping experiments was now led into a measuring cylinder which was held in a bucket of water (Figure 1D).

**Table 1:** Carbon dioxide flow rate (ml/min) produced by different yeast-sugar solutions

Application	Treatment	Average CO_2 _production (ml/min ± S.D.)
laboratory	7 g Y + 100 g S + 1 L W	3.5 ± 2.7
	70 g Y + 1000 g S + 10 L W (day 1)	14.1 ± 13.4
	70 g Y + 1000 g S + 10 L W (day 2)	62.6 ± 9.0

semi-field and field	17.5 g Y + 250 g S + 2.5 L W	136.3 ± 38.1
	17.5 g Y + 500 g S + 2.5 L W	242.3 ± 74.1
	17.5 g Y + 750 g S + 2.5 L W	144.8 ± 50.1
	35 g Y + 250 g S + 2.5 L W	220.2 ± 50.1
	35 g Y + 500 g S + 2.5 L W	303.5 ± 39.7
	35 g Y + 750 g S + 2.5 L W	298.1 ± 70.2

Averages are based on measurements taken each 15 or 30 minutes between 90 and 330 minutes (laboratory experiments) or 60 and 630 minutes (semi-field and field experiments) after mixing of the yeast-sugar solutions. Measurements were done indoors during the day at ambient temperature (22-25°C). Y: yeast; S: sugar; W: water.

Industrial carbon dioxide (≥ 99.9%) was released from pressurized gas cylinders (Linde Gas Benelux B.V., The Netherlands in laboratory experiments or Carbacid Investments Ltd., Kenya, in (semi-)field experiments) and supplied to the MM-X traps through silicon tubing (Ø 7 mm; Rubber B.V., The Netherlands). The Luer connection at the underside of the trap's top lid was used to release the gas directly into the odour outlet tube of the trap. A flow meter (Sho-Rate model GT1350 or GT1355, used in laboratory and semi-field experiments; Brooks Instruments, The Netherlands) or an orifice (American Biophysics Corp., USA; used in field experiments) regulated the flow rate of CO_2_. During the laboratory experiments, CO_2 _was led through a 0.5 L bottle before it was released into a MM-X trap. This bottle was filled for 50% with a 10% sugar solution.

Human foot odour was released from nylon socks (40 Den, 100% polyamide, HEMA, The Netherlands) worn by WHS (laboratory experiments) or KJvR (semi-field and field experiments) for 12 h prior to the experiments [6,12,13,26-29]. A clean nylon sock served as a control. Socks were placed along the odour outlet tube of the MM-X trap without blocking the airflow and held in position by odourless tape (3M™ Double Coated Tape 400; used in laboratory experiments) or by a small metal wire (in (semi-)field experiments).

### Laboratory experiments

Two MM-X traps were placed in a textile screen cage (330 × 250 × 233 cm; Howitec Netting BV, The Netherlands, [30]) at approximately 2.5 m distance from each other inside a climate-controlled room (22.2 ± 1.6°C and 52.6 ± 7.8% RH). The CO_2 _cylinder and the yeast-produced CO_2 _bottles were positioned within the sluice of the cage. Either two 1.5 L bottles or one 25 L container contained the yeast-sugar solution. In each 1.5 L bottle, 7 g of dry yeast and 100 g of refined household sugar were dissolved in 1 L of tap water. In the 25 L container, a mixture of 70 g of dry yeast, 1 kg of sugar and 10 L of tap water was prepared. In contrast to the 1.5 L bottles, which were used during a single experiment only, the 25 L container was used during two consecutive days without adding additional yeast, sugar or water. During the time it was not used, the container was closed and stored at room temperature. Worn socks were used to test the effect of human emanations on the attractiveness of yeast-produced CO_2_. The flow rate of industrial CO_2 _was set at 15 ml/min, a flow rate within the range (up to 20 ml/min) that was previously measured to be produced by two 1.5 L bottles each containing a mixture of 7 g of dry yeast, 100 g sugar and 1 L of tap water.

Experiments were conducted in the last 4 h of the dark phase when *An. gambiae *is normally searching for a blood host [31-33]. For each replicate, 50 mosquitoes were released from the centre of the screen cage and left in it for 4 h. After this period, the release cage and the traps were closed, the mosquitoes killed by freezing, and counted. The dual-choice experiments conducted are listed in Table 3. Treatments were alternated between the two positions to rule out any positional effect. In addition, experiments with two unbaited MM-X traps were conducted to test for positional effects. Each dual-choice experiment was replicated 6-8 times. Surgical gloves were worn by the operator to avoid contamination of equipment with human volatiles.

### Semi-field experiments

#### General

The semi-field experiments were conducted under ambient temperature and humidity (26.6 ± 0.9°C and 92.1 ± 8.9% RH) at the Thomas Odhiambo campus of ICIPE, Mbita Point, Kenya. Each semi-field experiment started at 9:30 pm by connecting the CO_2 _tubing and powering the traps, followed by releasing the mosquitoes. At 6:30 am the following morning the experiments were terminated by closing the traps and disconnecting the carbon dioxide and power supplies. The MM-X traps and collection bags of the CDC traps were placed in a freezer to kill the caught mosquitoes prior to counting. In addition, at 11 am the number of mosquitoes resting inside the house in the MalariaSphere was determined by way of actively searching for mosquitoes. In dual-choice MM-X experiments treatments were alternated between the two positions to rule out any positional effect. In addition, experiments with two unbaited MM-X traps were conducted to test for positional effects. Surgical gloves were worn to avoid contamination of equipment with human volatiles.

#### Effect of CO_2 _flow rate on trap catches

Experiments with industrial CO_2 _were conducted to establish the minimal CO_2 _flow rate needed to catch *An. gambiae *females using MM-X traps. For this purpose, a cage made of mosquito netting (2 × 2 × 6 m) was constructed inside a greenhouse (Cambridge Glass House Co. Ltd., UK) at Mbita Point, western Kenya. The greenhouse had a glass-panelled roof, gauze covered side walls, and sand on the floor [24,29]. Two MM-X traps were placed at opposite ends of the cage at a distance of approximately 5½ m of each other. Carbon dioxide was provided from a gas cylinder positioned outside the cage. During each experiment the CO_2 _cylinder was connected to one of the MM-X traps (for details see above). The other MM-X trap was unbaited. Five CO_2 _flow rates were tested: 25, 60, 100, 250 and 500 ml/min. These flow rates were chosen because they are commonly used to bait traps in mosquito surveillance exercises and/or are close to flow rates previously measured to be produced by the yeast-sugar solutions that had been tested in the laboratory. Each flow rate was tested four times. In each experiment 100 female mosquitoes were released from the centre of the cage.

#### Effect of yeast-produced CO_2 _on trap catches

Two MM-X traps were placed in the opposite corners of a screen-walled greenhouse (11.4 × 7.1 × 2.5 m, Cambridge Glass House Co. Ltd.) with a large mosquito-netting cage (10 × 6 × 2.5 m; mesh width 3 mm) suspended from the ceiling to the floor (screen house; [29]). This resulted in a distance of approximately 12½ m between the traps placed at 1½ m from the corner. A CO_2 _cylinder was placed next to each trap and CO_2 _was led to the trap using silicon tubing (for details see above). During the experiments traps were either unbaited, baited with industrial or yeast-produced CO_2 _or/and a worn sock. Industrial CO_2 _was applied at a flow rate of 100 or 250 ml/min. Yeast-produced CO_2 _was also applied at two different flow rates, using either a mixture of 17.5 g of dry yeast (Angel), 250 g sugar (Sony) and 2½ L of tap water or 35 g of dry yeast (Angel), 500 g sugar (Sony) and 2½ L of tap water in each 5 L bottle. The flow rates for industrial and yeast-produced CO_2 _were chosen based on the results obtained in the previously described experiments (see Table 4) and the flow rates measured to be produced by different yeast-sugar solutions (see Table 1), taking into account that temperatures are lower during the night than during the day, resulting in a lower production by the yeast-sugar solution.

In addition, the effectiveness of yeast-produced CO_2 _was tested 24 h and 48 h after mixing the ingredients. Each dual-choice experiment was done four times, each with 200 female mosquitoes released from the centre of the screen house. See Table 5 for an overview of the experiments performed.

#### Effect of CO_2 _baited traps on house entry behaviour

The MalariaSphere described by Knols et al [24] was used to test the potential of MM-X traps baited with either industrial or yeast-produced CO_2 _to reduce house entry by *An. gambiae *females [34,35]. The MalariaSphere consists of a screen-walled greenhouse (11.4 × 7.1 × 2.5 m, Cambridge Glass House Co. Ltd.) in which a traditional African house (3.2 × 2.8 × 1.7 m) has been built and crops planted.

**Figure 2:**
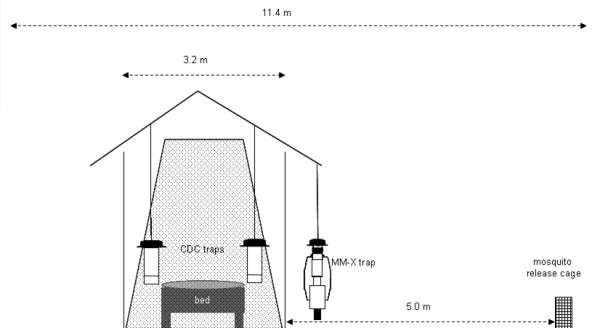
**Diagram showing the placement of the three traps inside (two CDC traps) and outside (a MM-X trap) an African house during the experiments conducted in the MalariaSphere **[24].

During the experiments, a male African volunteer (aged 27) slept inside the house on a bed, protected by an untreated bed net. Two CDC miniature light traps were hung at a height of 140 cm (bottom at 80 cm) above ground level beside the bed net on the foot-side end of the sleeping volunteer, with its shield touching the side of the bed net [36]. An odour-baited MM-X trap was hung outdoors under the overhanging part of the thatched roof of the house, 15 cm above ground level [12,13]. Either industrial CO_2 _at a flow rate of 100 ml/min or yeast-produced CO_2 _produced by 17.5 g dry yeast (Angel) + 250 g sugar (Sony) + 2½ L tap water in each 5 L bottle was tested. Also the effect of the addition of human emanations to CO_2 _was examined by putting a worn sock in the MM-X trap (see Table 6). Each treatment was tested six times, and in each experiment 200 female mosquitoes were released 5 m away from the house (Figure 2).

### Field experiments

The field experiments were conducted in Lwanda, a rural village at an altitude of 1169 m above sea level in the basin region of Lake Victoria, Nyanza Province, western Kenya. The area has a main rainy season from March to May and a short rainy season from October to December. Experiments were conducted at the end of the short rainy season, in December 2008. Lwanda has a variety of mosquito breeding habitats [37,38].

Based on several criteria (household, location of cooking site, roof construction, vegetation around the house and all houses at walking distance from each other) four approximately similar houses in Lwanda were selected. The occupants of the houses were sleeping under bed nets. Each house was provided with a MM-X trap, a car battery and a CO_2 _cylinder. The MM-X traps were hung outdoors, 15 cm above ground level, under the overhanging thatched roof, at the window side of the house [13]. Vaseline petroleum jelly was used around the tubing, suspension cable and electrical cables to prevent ants from reaching the mosquitoes caught in the MM-X trap.

Two series of each eight nights (i.e. two blocks of a 4 × 4 Latin square) were run. In the first series the following four treatments were tested: unbaited, industrial CO_2 _at a flow rate of 250 ml/min (the amount of CO_2 _released by a human, [2]), and yeast-produced CO_2 _at two different flow rates, using either a mixture of 17.5 g of dry yeast (Angel), 250 g sugar (Sony) and 2½ L of tap water or 35 g of dry yeast (Angel), 500 g sugar (Sony) and 2½ L of tap water in each 5 L bottle. In the second series the effect of the combination of CO_2 _and human emanations on the trap catches was examined by testing industrial CO_2 _at a flow rate of 250 ml/min with or without the addition of a worn sock, and yeast-generated CO_2 _produced by 35 g of dry yeast (Angel), 250 g sugar (Sony) and 2½ L of tap water in each 5 L bottle with or without the addition of a worn sock. Each experiment ran from 8:30 pm until 6:30 am, after which the mosquitoes in the traps were killed by placing the traps in a freezer and counted. Surgical gloves were worn to avoid contamination of equipment with human volatiles.

The mosquitoes caught in each trap during one night were morphologically identified and counted. Culicines were identified to genus, anophelines to species. Female *An. gambiae sensu lato *mosquitoes were placed in a 2 ml Eppendorf tube with dry silica gel and a piece of cotton wool. These mosquitoes were transported to the Laboratory of Entomology of Wageningen University for species identification. The Bender buffer method [39] was used to extract DNA from a mosquito leg and part of the abdomen of each mosquito, followed by polymerase chain reaction (PCR) analysis [40].

### Yeast-produced CO_2 _concentration measurements

The concentration of yeast-generated CO_2 _produced by 17.5 g of dry yeast, 250 g sugar and 2½ L of tap water in each 5 L bottle and flowing from a MM-X trap was measured in the laboratory using a Xentra 4100 CO_2 _analyser (Servomex, The Netherlands). The data were transferred to a PC using Das Wizard 2.0 software (Measuring Computing Corporation, USA). The analyser measured at 1 Hz and was programmed to shift to the next measuring point after 60 successive readings. The average of these 60 readings was plotted in a graph. The analyzer measured over a range of 0-1030 ppm with an accuracy of 0.1 ppm.

Three series of readings, each for a duration of 20 min, were taken at different times after mixing the yeast-sugar solution (1½, 25½ and 49½ h). For each series of readings, three measuring points were positioned at different distances from the MM-X trap (Table 2). To make a comparison with concentrations flowing from a MM-X trap baited with industrial CO_2 _another three series of readings, at different distances from the MM-X trap, were made (Table 2). For this comparison the human equivalent of CO_2 _percentage present in breath (5%) and the amount released (250 ml/min) were chosen [1,2].

**Table 2:** Position of CO_2 _measurements; CO_2 _either produced by a yeast-sugar solution (17.5 g yeast+250 g sugar+2½ L water in each 5 L bottle) or released from a CO_2 _cylinder (5%, 250 ml/min)

Measuring points	Series 1	Series 2	Series 3
1	inside MM-X outlet	10 cm below MM-X^1^	above MM-X^2^
2	10 cm below MM-X^1^	30 cm from MM-X^1^	30 cm from MM-X^2^
3	200 cm from MM-X^1^	200 cm from MM-X^1^	200 cm from MM-X^2^

^1 ^Measuring point positioned 5 cm above ground level

^2 ^Measuring point positioned 100 cm above ground level

### Statistical analysis

For each dual-choice test (laboratory and semi-field experiments) a χ^2^-test was used to test whether the distribution of the total number of mosquitoes caught in the treatment or control trap over all replicates differed from a 1:1 distribution. A χ^2^-test was also used to compare the total number of mosquitoes found inside (total number caught by the two CDC light traps + found resting inside the house) and caught outside (by the MM-X trap) the house in the MalariaSphere. Effects were considered to be significant when P < 0.05.

Of the mosquitoes caught during the field experiments, the rarely caught male mosquitoes were discarded from the data. Due to many zeros, the numbers of anopheline and *Aedes *females were transformed (natural logarithm of (x+1)) before subjection to a Generalized Linear Model (GLM; Genstat^®^ release 12.1; Normal distribution, fitted terms: night, house, treatment, and when significant the interaction between house and treatment). *Mansonia*, *Culex *and total counts were not transformed before subjection to a GLM (Poisson distribution, linked in log, dispersion estimated to account for heterogeneity, fitted terms: night, house, treatment, and when significant the interaction between house and treatment). Two-sided t-probabilities were calculated to test pairwise differences of means. Effects were considered to be significant when P < 0.05.

## Results

### Laboratory experiments

Experiments with two unbaited MM-X traps revealed no positional effect within the cage (P = 0.24, n = 297; Table 3). In total, 15.5% of the mosquitoes were caught by the two traps. A trap baited with a worn sock caught significantly more mosquitoes than a trap baited with a clean sock (P < 0.001, n = 277). The two traps together caught on average 41.6% of the mosquitoes that flew out of the release cage.

**Table 3:** Effect of yeast-produced CO_2 _on trap catches during laboratory experiments

Treatment	Control	Total number caught		χ^2^-test	N	Mean % caught (± sem)^1^
					
		T	C			
no odour	no odour	19	27	P = 0.24	297	15.5 ± 3.0
worn sock	clean sock	96	21	P < 0.001	277	41.6 ± 7.9
yeast CO_2 (7 g)_	no odour	186	29	P < 0.001	279	77.0 ± 7.3
yeast CO_2 (7 g)_	ind. CO_2 (15 ml/min)_	103	51	P < 0.001	298	51.6 ± 8.5
yeast CO_2 (7 g) _+ worn sock	worn sock	96	62	P = 0.007	278	55.5 ± 12.1
yeast CO_2 (70 g) _+ worn sock	worn sock	207	93	P < 0.001	371	78.8 ± 10.8

Yeast = yeast-produced (either 7 g yeast+100 g sugar+1 L water in each 1.5 L bottle or 70 g yeast+1 kg sugar+10 L water in a 25 L container)

Ind. = industrial (15 ml/min)

T = treatment

C = control

N = total number of mosquitoes released

^1 ^Mean percentage caught expressed as the number of female mosquitoes caught in the two MM-X traps together divided by the number of mosquitoes that flew out of the release cage.

Underlined number: significant higher catch (χ^2^-test, P < 0.05)

A trap baited with yeast-produced CO_2_, produced by a mixture of 7 g of dry yeast, 100 g sugar and 1 L of tap water in each 1.5 L bottle, caught significantly more mosquitoes than an unbaited trap (P < 0.001, n = 279). During these experiments, on average 77.0% of the mosquitoes released were caught. Also when the other trap was baited with industrial CO_2 _(15 ml/min) led through two 1.5 L bottles each filled with 1 L of sugar water, the trap baited with yeast-produced CO_2 _(two 1.5 L bottles with each 7 g dry yeast + 100 g sugar + 1 L water) caught significantly more mosquitoes (P < 0.001, n = 298, in total 51.6% caught).

Significantly more mosquitoes were caught by traps baited with yeast-produced CO_2 _combined with a worn sock than traps baited with a worn sock only. This was observed when two 1.5 L bottles each containing 7 g dry yeast+100 g sugar+1 L water were used for the production of yeast-produced CO_2 _and when one 25 L container with 70 g of dry yeast, 1 kg of sugar and 10 L of tap water was used (P = 0.007, n = 278 respectively P < 0.001, n = 371). In total a mean of 55.5% and 78.8%, respectively, of the mosquitoes that had left the release cage were caught during these experiments.

### Semi-field experiments

#### Effect of CO_2 _flow rate on trap catches

No positional effects were found in the cage when both traps were left unbaited (P = 0.33, n = 200; Table 4); the two unbaited traps together caught on average 19.0% of the mosquitoes released. A trap baited with 25 ml/min of industrial CO_2 _caught similar numbers of mosquitoes as an unbaited trap (P = 0.07, n = 400). In total, a mean of 37.5% of the mosquitoes was trapped. Traps baited with industrial CO_2 _at a flow rate of 60, 100, 250 or 500 ml/min caught significantly more mosquitoes than unbaited traps (P < 0.001, < 0.001, < 0.001 and 0.03, respectively, n = 400). The traps caught on average 30.5, 54.8, 39.5, and 29.5% of the females that left the release cage, respectively.

**Table 4:** Effect of CO_2 _flow rate on trap catches during screen house experiments

CO_2 _flow rate (ml/min)	Total number caught		χ^2^-test	N	Mean % caught (± sem)^1^
				
	ind. CO_2_	no odour			
0	22	16	P = 0.33	200	19.0 ± 3.0
25	86	64	P = 0.07	400	37.5 ± 6.1
60	100	22	P < 0.001	400	30.5 ± 8.0
100	177	42	P < 0.001	400	54.8 ± 7.6
250	126	32	P < 0.001	400	39.5 ± 4.9
500	71	47	P = 0.03	400	29.5 ± 4.6

Ind. = industrial

N = total number of mosquitoes released

^1 ^Mean percentage caught expressed as the number of female mosquitoes caught in the two MM-X traps together divided by the number of mosquitoes that flew out of the release cage.

Underlined number: significant higher catch (χ^2^-test, P < 0.05)

#### Effect of yeast-produced CO_2 _on trap catches

Experiments in the screen house with unbaited traps revealed no bias for either side (P = 0.64, n = 800; Table 5). The two unbaited traps together caught only 5.1% of the mosquitoes that were released. A worn sock attracted significantly more mosquitoes than a clean sock (P < 0.001, n = 800); on average 43.1% of the mosquitoes were trapped.

**Table 5:** Effect of yeast-produced CO_2 _on trap catches during screen house experiments

Treatment	Control	Total number caught		χ^2^-test	N	Mean % caught (± sem)^1^
					
		T	C			
no odour	no odour	22	19	P = 0.64	800	5.1 ± 0.7
worn sock	clean sock	288	48	P < 0.001	800	43.1 ± 4.1
yeast CO_2 (17.5 g)_	no odour	250	16	P < 0.001	800	33.3 ± 5.7
yeast CO_2 (35 g)_	no odour	251	11	P < 0.001	800	32.8 ± 5.1
yeast CO_2 (17.5 g)_	ind. CO_2 (100 ml/min)_	274	190	P < 0.001	800	58.0 ± 7.9
yeast CO_2 (35 g)_	ind. CO_2 (250 ml/min)_	326	244	P < 0.001	800	71.3 ± 2.6
yeast CO_2 (17.5 g) _+ worn sock	clean sock	411	13	P < 0.001	800	53.0 ± 12.7
yeast CO_2 (17.5 g) _+ worn sock	worn sock	581	55	P < 0.001	800	79.5 ± 2.5
yeast CO_2 (17.5 g) _+ worn sock	ind. CO_2 (100 ml/min) _+ worn sock	342	265	P = 0.002	800	75.9 ± 1.8

yeast CO_2 (17.5 g) _24 h	no odour	132	18	P < 0.001	800	18.8 ± 2.9
yeast CO_2 (17.5 g) _24 h	ind. CO_2 (100 ml/min)_	240	384	P < 0.001	800	78.0 ± 4.7
yeast CO_2 (17.5 g) _48 h	no odour	57	41	P = 0.11	800	12.3 ± 1.9
yeast CO_2 (17.5 g) _48 h	ind. CO_2 (100 ml/min)_	30	342	P < 0.001	800	46.5 ± 2.8

Yeast = yeast-produced (either 17.5 g yeast+250 g sugar+2½ L water or 35 g yeast+500 g sugar+2½ L water in each 5 L bottle)

Ind. = industrial (flow rate given in ml/min)

T = treatment

C = control

N = total number of mosquitoes released

^1 ^Mean percentage caught expressed as the number of female mosquitoes caught in the two MM-X traps together divided by the number of mosquitoes that flew out of the release cage.

Underlined number: significant higher catch (χ^2^-test, P < 0.05)

Significantly more mosquitoes were caught by traps baited with yeast-produced CO_2 _than unbaited traps, independent of the ratio used for the yeast-sugar solution (P < 0.001, n = 800). Traps baited with yeast-produced CO_2 _also caught significantly more mosquitoes when tested against traps baited with industrial CO_2_, independent of the flow rate tested (P < 0.001, n = 800). On average, between 32.8 and 71.3% of the females were caught (Table 5).

As expected, traps baited with the combination of yeast-produced CO_2 _(17.5 g of dry yeast (Angel), 250 g sugar (Sony) and 2½ L of tap water in each 5L bottle) and a worn sock caught significantly more mosquitoes than unbaited traps (P < 0.001, n = 800), catching in total 53.0% of the released mosquitoes (Table 5). This combination attracted also significantly more mosquitoes than a worn sock alone (P < 0.001, n = 800), resulting in a total trapping efficacy of 79.5%. Significantly fewer mosquitoes were caught by traps baited with a combination of industrial CO_2 _(100 ml/min) and a worn sock than traps baited with yeast-produced CO_2 _and a worn sock (P = 0.002, n = 800, in total 75.9%).

Twenty-four hours after mixing the ingredients, significantly more mosquitoes were trapped using yeast-produced CO_2 _than when no bait was used (P < 0.001, n = 800), catching a mean total of 18.8% (Table 5). However, significantly more mosquitoes were caught by traps baited with industrial CO_2 _(100 ml/min) than by traps baited with yeast-produced CO_2 _prepared 24 h before the start of the dual-choice trapping experiments (P < 0.001, n = 800; 78.0%). After 48 h, traps baited with yeast-produced CO_2 _caught similar numbers of mosquitoes as unbaited traps (P = 0.11, n = 800, 12.3%), and significantly fewer mosquitoes than traps baited with industrial CO_2 _(P < 0.001, n = 800, 46.5%).

#### Effect of CO_2_-baited traps on house entry behaviour

The number of mosquitoes trapped by a MM-X trap baited with yeast-produced CO_2 _hanging outside the house in the MalariaSphere was significantly higher than the total number of mosquitoes that entered the house when unoccupied (total number caught by the two CDC light traps + found resting inside the house) (P < 0.001, n = 800; Table 6). In total, 58.5% of the mosquitoes that were released were either caught by the three traps (one MM-X, two CDC light traps) or found resting inside the house. In contrast, when the house was occupied by a human sleeping under a bed net, significantly more mosquitoes entered the house than were caught by the yeast-produced CO_2_-baited MM-X trap (P < 0.001, n = 800). This was also the case when the MM-X trap was baited with industrial CO_2 _(100 ml/min; P < 0.001, n = 800). Together, 47.8% (yeast-produced), respectively 53.5% (industrial) of the mosquitoes were retrieved, 'outdoors' plus 'indoors'.

**Table 6:** Effect of CO_2 _baited traps on house entry behaviour in the MalariaSphere

Treatment outdoors	Treatment indoors	Total number caught		χ^2^-test	N	Mean % Caught (± sem)^1^
					
		O	I			
yeast CO_2 (17.5 g)_	no odour	367	101	P < 0.001	800	58.5 ± 5.7
yeast CO_2 (17.5 g)_	human	115	267	P < 0.001	800	47.8 ± 6.2
ind. CO_2 (100 ml/min)_	human	169	259	P < 0.001	800	53.5 ± 5.5
yeast CO_2 (17.5 g)_+ worn sock	human	454	96	P < 0.001	800	68.8 ± 9.3
ind. CO_2 __(100 ml/min)_+ worn sock	human	407	184	P < 0.001	800	73.9 ± 7.5

Yeast = yeast-produced (17.5 g yeast+250 g sugar+2½ L water)

Ind. = industrial (100 ml/min)

O = number of mosquitoes caught in the MM-X trap outside the house

I = total number of mosquitoes caught in 2 CDC light traps and found resting inside the house

N = total number of mosquitoes released

^1 ^Mean percentage caught expressed as the number of female mosquitoes recovered from inside the three traps (1 MM-X, 2 CDC) and the house together divided by the number of mosquitoes that flew out of the release cage.

Underlined number: significant higher catch (χ^2^-test, P < 0.05)

When a worn sock was added to the MM-X trap baited with either yeast-produced or industrial CO_2_, significantly more mosquitoes were trapped outdoors than caught in the CDC traps and found resting indoors where a human was present (P < 0.001, n = 800): of all mosquitoes trapped, 68.9% (with industrial CO_2_) to 82.5% (with yeast-produced CO_2_) were caught in the CO_2 _+ human odour baited MM-X trap. In total, 68.8% (yeast-produced), respectively 73.9% (industrial) of the mosquitoes released were recovered from inside the three traps and the house together.

### Field experiments

In the first series of field experiments 392 and in the second series 486 female mosquitoes were caught over eight nights in traps hanging next to the four selected houses. The majority consisted of *Mansonia *spp. mosquitoes: 48.7% and 66.0% in series 1 and 2, respectively. Also *Culex *spp. females were caught in high proportions: 34.7% respectively 23.3% of the total number of female mosquitoes found in the traps. Of the anophelines (12.2% and 9.7%, respectively) 3.8% respectively 5.1% were *An. gambiae s.l*. females. PCR tests revealed that all (except five specimens that could not be identified) of the *An. gambiae s.l*. specimens were *Anopheles arabiensis*. The majority of the anophelines were *Anopheles coustani *females; only a few *Anopheles funestus *(1%) were found in the traps. In addition, 4.3% respectively 1.0% of the mosquitoes caught were *Aedes *spp.

GLM analysis showed that both in series 1 and 2 the average number of mosquitoes caught by the four traps hardly varied during the eight nights, whereas the location of the trap (i.e. house) often significantly affected the number of mosquitoes trapped during a night (Table 7). In the first series a significant effect of treatment was found for *Culex *and *Mansiona *spp., as well as for *Culex *spp. in the second series.

**Table 7:** Mean ± SD mosquitoes caught during field experiments by MM-X traps baited with different test odours

Series	Test odour	*An. coustani*	*An. funestus*	***An. gambiae s.l***.	**tot. anoph**.	***Aedes *spp**.	***Culex *spp**.	***Mansonia *spp**.	**tot. non-anoph**.	tot. mosquitoes
1	no odour	0 ± 0 a	0 ± 0 a	0 ± 0 a	0 ± 0 a	0.4 ± 0.5 a	1.3 ± 1.5 a	0.5 ± 0.8 a	2.1 ± 1.8 a	2.1 ± 1.8 a
	
	ind. CO_2 __(250 ml/min)_	1.3 ± 2.8 ab	0.1 ± 0.4 a	1.0 ± 2.1 b	2.4 ± 4.8 ab	0.6 ± 0.7 a	7.9 ± 6.9 ab	10.3 ± 7.5 c	18.8 ± 12.6 c	21.1 ± 17.1 c
	
	yeast CO_2 (17.5 g)_	0.6 ± 0.7 ab	0.1 ± 0.4 a	0.4 ± 1.1 ab	1.1 ± 1.6 ab	0.1 ± 0.4 a	2.3 ± 2.1 a	4.9 ± 6.5 b	7.3 ± 6.3 b	8.4 ± 6.9 b
	
	yeast CO_2 (35 g)_	1.6 ± 1.9 b	0.4 ± 1.1 a	0.5 ± 1.1 ab	2.5 ± 2.9 b	0.5 ± 1.4 a	5.6 ± 6.7 b	8.3 ± 7.9 bc	14.9 ± 14.6 c	17.4 ± 14.0 c

P_night_		0.80	0.17	0.16	0.76	0.29	0.43	0.17	0.15	0.26
		
P_house_		0.17	0.69	0.01*	0.04	0.87	0.11	0.02	0.01	0.002
		
P_treatm_		0.14	0.69	0.12*	0.07	0.28	0.007	< 0.001	< 0.001	< 0.001

2	ind. CO_2 __(250 ml/min)_	0.6 ± 1.1 ab	0 ± 0 a	0 ± 0 a	0.6 ± 1.6 a	0.3 ± 0.5 a	4.4 ± 3.9 b	7.3 ± 5.2 a	11.9 ± 8.1 ab	12.5 ± 8.9 ab
	
	ind. CO_2 __(250 ml/min) _+ worn sock	0.8 ± 0.7 b	0.3 ± 0.5 a	1.3 ± 1.8 b	2.3 ± 2.7 b	0.3 ± 0.5 a	5.4 ± 6.0 b	12.9 ± 7.8 a	18.5 ± 10.1 b	20.8 ± 11.9 b
	
	yeast CO_2 (35 g)_	0.8 ± 1.0 b	0.1 ± 0.4 a	0.4 ± 1.1 a	1.3 ± 2.1 ab	0.1 ± 0.4 a	2.8 ± 4.4 a	8.1 ± 6.5 a	11.0 ± 7.8 a	12.3 ± 9.2 a
	
	yeast CO_2 (35 g) _+ worn sock	0 ± 0 a	0.3 ± 0.7 a	1.5 ± 1.6 b	1.8 ± 2.3 ab	0 ± 0 a	1.6 ± 1.7 a	11.9 ± 8.6 a	13.5 ± 8.1 ab	15.3 ± 9.9 ab

P_night_		0.22	0.47	0.32	0.83	0.89	0.001	0.29	0.39	0.44
		
P_house_		0.02	0.004	< 0.001*	0.001	0.58	< 0.001	0.008	0.004	0.003
		
P_treatm_		0.05	0.46	0.001*	0.18	0.58	0.003	0.15	0.17	0.16

Yeast = yeast-produced (17.5 g yeast+250 g sugar+2½ L water or 35 g yeast+500 g sugar+2½ L)

Ind. = industrial (250 ml/min)

tot. = total

anoph. = anophelines

Mean numbers caught marked with different letters within a column within same series are significantly different (GLM, P < 0.05)

* interaction between house and treatment (GLM, P = 0.04 in series 1 and P = 0.03 in series 2)

In the case of *An. gambiae s.l*., the effect of the different baits (treatment) on the number of mosquitoes caught depended on the location of the trap (P_interaction _= 0.04 in series 1 and P_interaction _= 0.03 in series 2). In the first series, traps baited with industrial CO_2 _caught significantly more *An. gambiae s.l*. than unbaited traps (P = 0.02), but similar numbers as traps baited with yeast-produced CO_2 _(P = 0.14 and 0.33, respectively; Table 7). The second series of experiments showed that, overall, adding a worn sock to either yeast-produced or industrial CO_2 _significantly increased the number of mosquitoes caught (P = 0.003 and 0.002, respectively). Traps baited with yeast-produced CO_2 _plus a worn sock also caught more mosquitoes than industrial CO_2 _alone (P = 0.02). The majority of the *An. gambiae s.l*. females were trapped next to house #1 (P < 0.05).

Taking all mosquito species caught during the first series together, unbaited traps caught significantly fewer mosquitoes than odour-baited traps (P < 0.05). Traps baited with yeast-produced CO_2 _at the lowest flow rate caught significantly fewer mosquitoes than traps baited with yeast-produced CO_2 _at the highest flow rate and traps baited with industrial CO_2 _(P = 0.009 and 0.003, respectively). Traps baited with the latter two baits caught similar numbers of mosquitoes (P = 0.74). In the second series the location of the traps determined the total numbers of mosquitoes caught (P = 0.003), independent of treatment.

### Yeast-produced CO_2 _concentration measurements

**Figure 3:**
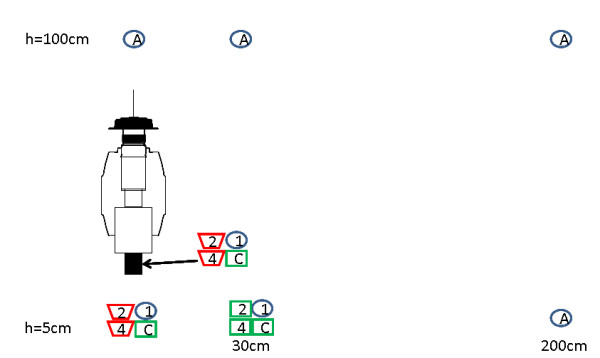
**Diagram summarising industrial and yeast-produced CO_2 _concentrations measured at different distances of a MM-X trap**. Blue circle: 400-500 ppm; green rectangular: 500-600 ppm; red triangle: > 600 ppm; 1, 2 and 4: 1½, 25½ and 49½ h post mixing the yeast-sugar solution (17.5 g yeast+250 g sugar+2½ L water in each 5 L bottle); C: industrial CO_2 _(5%, 250 ml/min); A: all (yeast-produced and industrial) CO_2 _sources.

The carbon dioxide concentrations measured at different distances from a MM-X trap are summarized in Figure 3. It shows clearly the distance effect on the concentration of CO_2_, the further away from the MM-X trap the lower the CO_2 _concentration, independent of its source (CO_2 _cylinder or yeast-sugar solution 1½, 25½ or 49½ h post mixing). Concentrations measured at a distance of 200 cm or at a height of 100 cm were between 400 and 500 ppm. Measurements taken 1½ hours after mixing the yeast-sugar solution, within or close to the trap (0 and 30 cm from the trap, 5 cm above ground level) also showed CO_2 _levels between 400 and 500 ppm.

Carbon dioxide concentrations produced by yeast-sugar solutions 25½ or 49½ h post mixing, measured inside or 10 cm below the trap outlet, was higher (600-850 ppm) than when industrial CO_2 _(5%, 250 ml/min) was used to bait the trap (500-600 ppm). At 30 cm from the trap and 5 cm above ground level, CO_2 _levels were similar for industrial and yeast-sugar solutions 25½ and 49½ h after mixing (450-550 ppm).

## Discussion

Based on the results, CO_2_, and possibly other volatiles, produced by fermenting baker's yeast appears a promising alternative for industrial CO_2 _supplied from expensive and cumbersome cylinders to lure *An. gambiae *females towards traps. Trap catches were similar or even significantly higher when yeast-produced CO_2 _was used to bait MM-X traps compared to industrial CO_2_. This finding presents an important step in the development of a cheap and easily applicable CO_2 _source that could be used for mosquito surveillance or removal in rural settings.

The indoor and semi-field trapping experiments showed that yeast-produced CO_2_, produced by yeast-sugar solutions in different ratios, significantly increased the number of *An. gambiae *females caught by MM-X traps. Traps baited with yeast-produced CO_2 _also caught significantly more mosquitoes than traps baited with industrial CO_2 _at a similar or probably higher flow rate. Yeast-produced CO_2 _also significantly increased the catches of traps baited with human odour collected on nylon socks (Tables 3 and 5). These finding are in agreement with previous research with industrial CO_2_, showing the importance of this compound in the trapping of this mosquito species [5,11-13].

The experiments conducted in the MalariaSphere revealed that a trap baited with yeast-produced CO_2 _hanging outdoors next to a house caught significantly more *An. gambiae *than entered the unoccupied house. This was not the case when a human was sleeping inside the house, regardless of the use of yeast-produced or industrial CO_2 _as only bait in a trap placed outdoors. However, when adding human foot volatiles to either yeast-produced or industrial CO_2_, significantly fewer mosquitoes were found inside the occupied house than in the MM-X trap placed under the eaves outdoors (Table 6), proving that the combination of human skin odour + CO_2 _effectively prevented a large proportion of mosquitoes entering the house. These encouraging results suggest that it is possible to develop traps that can be placed outdoors, baited with CO_2 _and a synthetic blend mimicking human odour, to reduce the number of malaria mosquitoes entering houses through the eaves. Jawara *et al *[13], however, showed that in The Gambia human odour-baited traps placed either next to or inside an experimental house did not decrease the number of wild mosquitoes entering the house. Other measures, like house screening or application of repellent odours, to prevent house entry may therefore be necessary to apply in addition to odour-baited traps [41]. Semi-field and field experiments are ongoing to explore this further.

During the field experiments in the present study, mosquito numbers were low and no *An. gambiae s.s*., the subject of our study, was caught. Its sibling species, *An. arabiensis*, however, was present and collected significantly more with human skin odour + CO_2 _than with CO_2 _alone (Table 7). Similar comparative results for *An. gambiae s.s*. and *An. arabiensis *with odour baits were also reported by Okumu *et al *[42], suggesting that both important malaria vectors can be collected with odour-baited traps. Also, yeast-produced CO_2 _seems to be as good as industrial CO_2 _as bait for several other vector and nuisance mosquito species (Table 7).

In the laboratory and screen house significantly more mosquitoes were caught in traps baited with yeast-produced CO_2 _than in traps baited with industrial CO_2 _when tested directly against each other. Since the flow rates were either comparable or more likely lower for yeast-produced CO_2 _(e.g., due to lower temperatures during the night), possible differences in flow rates between industrial and yeast-produced CO_2 _did not result in differences in attractiveness. It is, however, known that growing yeast produces additional compounds besides CO_2 _[20]. Preliminary analyses of headspaces of yeast-sugar solutions (70 g Y + 1000 g S + 10 L W in 25 L container), two and 28 h post mixing, revealed that yeast produces volatile organic compounds (VOCs) previously found in human emanations and which may therefore play a role in the host-seeking behaviour of *An. gambiae s.s*. [43-46] (Table 8). These additional VOCs may explain the differences found in catches between traps baited with yeast-produced CO_2 _compared to traps baited with industrial CO_2 _and should be further examined.

**Table 8:** Preliminary data of volatile organic compounds found to be more present in headspace samples of yeast-sugar solutions (2 or 28 h post mixing) than in background samples (order of compounds based on retention time on a DB-5 column)

Compound	Yeast sample	Human emanation references
ethanol	2 h, 28 h	[54-56]
2-methylpropanal	2 h	[57]
ethyl acetate	2 h, 28 h	[56]
2-methyl-1-propanol	2 h, 28 h	[54]
3-methylbutanal	28 h	[46,58,59]
1-pentanol	28 h	[55]
3-hydroxy-2-butanone	2 h	[46,55]
3-methyl-1-butanol	2 h, 28 h	[46,55]
2-methylpropanoic acid	2 h, 28 h	[60,61]
3-methylbutanoic acid	2 h	[46,55,60-62]
benzeneethanol	2 h, 28 h	[46,57,62,63]
isobutyl ester of ethanoic acid	28 h	
ethyl 2-methylbutanoate	28 h	[55]
ethyl 3-methylbutanoate	28 h	[55]
3-methylbutyl acetate	28 h	
2-methylbutyl acetate	28 h	
ethyl ester of hexanoic acid	28 h	
1-dodecene	28 h	[63]
ethyl ester of octanoic acid	28 h	
ethyl ester of decanoic acid	28 h	
isopentyl ester of octanoic acid	28 h	

Measurements of CO_2 _concentrations at different distances from a MM-X trap showed that, at close range of the trap, CO_2 _concentrations produced by yeast-sugar solutions were higher than from cylinders containing 5% CO_2 _(equal to the concentration in human breath). Further away from the trap, at 30 cm, concentrations of industrial and yeast-produced CO_2 _had dropped to a comparable low level (Figure 3). Even though this was measured in a laboratory where no wind was present, it is very likely that also in the field packets of CO_2 _are produced by yeast-sugar solutions with concentrations similar to or higher than what is produced by humans [47-50]. Since mosquitoes respond to small changes in CO_2 _concentration above ambient, this will be sufficient to induce upwind flight [1,50-52].

In Japan and Malaysia, traps baited with dry ice caught more *Culex *and *Aedes *mosquitoes than traps baited with yeast-produced CO_2 _[16,53]. However, the advantages, such as low costs and feasible logistics, of the yeast-method clearly outweigh the logistic disadvantages and relatively high costs associated with both dry ice and CO_2 _cylinders. Variable CO_2 _output may occur when using yeast-sugar solutions, probably depending on the ambient temperature. This issue, however, is not problematic, since the current results show that mosquitoes are attracted to yeast-produced CO_2_, regardless of the concentrations used. In addition, indications have been found that fluctuating concentrations of CO_2 _above the ambient level induce upwind orientation of mosquitoes [50,52], although the laboratory and field experiments of the present study indicate that higher concentrations are favourable.

Both laboratory and semi-field experiments showed that yeast-produced CO_2 _is still 'attractive' 24-34 h post mixing the ingredients (Tables 3 and 5), although less than industrial CO_2 _(which is released with a constant flow rate and concentration), showing that this bait is at least applicable during one sampling night. In the screen house, yeast-produced CO_2 _lost its attractiveness somewhere between 34 and 48 h post-mixing the ingredients (Table 5). Carbon dioxide flow rates dropped from 60 ml/min after 30 h to 0 ml/min within 51 h. In contrast, the CO_2 _measurements showed that even after 49 h CO_2 _concentrations should be sufficiently high to activate mosquitoes (Figure 3) and simultaneous CO_2 _output measurements showed a flow rate of 30 ml/min. These differences may have been due to temperature differences or tap water of different sources.

## Conclusion

Carbon dioxide and possibly additional volatiles produced by yeast-sugar solutions are attractive to *An. gambiae *and, therefore, these solutions can be used as baits for the surveillance or possibly removal of this important malaria vector. The results suggest that CO_2 _is the most important constituent of these VOCs, because addition of human foot volatiles enhanced attraction of mosquitoes similar as with industrial CO_2_. As long as CO_2 _production will be sufficient for at least one night, the smaller the bottle and the cheaper and easier accessible the ingredients, the better for implementation in rural areas. This technology could represent a new solution for sampling *An. gambiae *and other human-biting mosquito species in remote areas, with low financial and technological demands.

## Competing interests

The authors declare that they have no competing interests.

## Authors' contributions

WHS conceived of the idea to test yeast as a carbon dioxide source to trap malaria mosquitoes. The experimental set-up was developed by WHS, KJR, WRM and RCS. WHS and KJR conducted the behavioural experiments, with the assistance of NOV. JS performed the carbon dioxide concentration measurements. RCS analysed the data and drafted the manuscript. All authors contributed to, read and approved the final manuscript.
